# Integrated assessment of rabies vaccination coverage and behavioral classification of pet owners using knowledge, attitude, and practice - based multivariate analytics in Duc Hue District, Vietnam (2024)

**DOI:** 10.14202/vetworld.2025.4146-4156

**Published:** 2025-12-31

**Authors:** Loan Phung Bich Tran, Bao Dinh Truong, Dien Thi Kieu Nguyen, Nhu Y Le Ngo, Tan Nhat Nguyen, Tuyet Anh Lam, Thinh Phuc Pham, Minh Duong Vo, Khanh Tran Vinh Doan, Oanh Thi Kieu Vo, Khanh Thi Mai Nguyen, Khanh My Thuy Bui, Thanh Ngoc Vo, Thong Quang Le

**Affiliations:** 1 Faculty of Animal Science and Veterinary Medicine, Nong Lam University, Ho Chi Minh City 71308, Vietnam; 2 Sub-Department of Animal Health of Tay Ninh Province, Vietnam; 3 Boehringer Ingelheim Company, Ho Chi Minh City, Vietnam

**Keywords:** cluster analysis, knowledge, attitude, practice, pet owner behavior, rabies control, rabies vaccination coverage, Vietnam, veterinary public health, zoonotic disease prevention

## Abstract

**Background and Aim:**

Rabies remains a fatal zoonotic disease of major public health importance in Vietnam, with rising human and animal cases in recent years. Achieving effective control requires high vaccination coverage in dogs and cats, as well as a clear understanding of the behavioral factors influencing vaccination decisions. This cross-sectional analytical study aimed to assess vaccination coverage, examine determinants of proactive vaccination behaviors, and classify pet owners based on their knowledge, attitude, and practice (KAP) regarding rabies prevention in Duc Hue District, Long An Province, during the 2024 mass vaccination campaign.

**Materials and Methods:**

The study was conducted across 11 communes between January 10 and April 25, 2024. Data collection included a general household survey and a structured KAP questionnaire. Digital tools such as KoboToolbox, QGIS version 3.36, and RStudio version 4.5.1 were used for data entry, mapping, and statistical analysis. Logistic regression identified demographic and logistical variables associated with proactive vaccination behavior. Principal component analysis (PCA) and cluster analysis (CA) were used to classify pet owners into behavioral groups based on KAP score patterns. Ethical approval was obtained from Nong Lam University and the Long An People’s Committee.

**Results:**

A total of 6,899 of 8,776 pets were vaccinated, achieving a coverage rate of 78.6%, surpassing the national target of 70% for 2022–2025, although coverage varied widely across communes (60.63%–87.78%). Logistic regression revealed that higher education levels, specific occupations, shorter distance to veterinary services, and smaller total pet populations were significant predictors of proactive vaccination behavior. PCA and CA identified three distinct groups of pet owners. The group with higher education levels and farming occupations demonstrated the strongest KAP profile, while the lowest-performing group was associated with limited education and greater logistical challenges in accessing veterinary services.

**Conclusion:**

This study presents the first integrated behavioral–statistical classification of pet owners in southern Vietnam using PCA and clustering, providing a data-driven foundation for more targeted rabies vaccination interventions. Addressing logistical barriers, improving equitable access to veterinary services, and tailoring educational activities toward low-performing groups are essential for sustaining vaccination gains and advancing Vietnam’s goal of achieving 80% coverage by 2030.

## INTRODUCTION

Rabies is a fatal zoonotic disease and one of the World Health Organization (WHO)-listed Neglected Tropical Diseases caused by the rabies virus (RABV), which belongs to the genus Lyssavirus and the family Rhabdoviridae. RABV is a multi-host pathogen that infects all warm-blooded animals; however, dogs, wild carnivores such as foxes, jackals, wolves, and mongooses, raccoons, badgers, and bats serve as the primary natural reservoirs of RABV. These animals are a major source of rabies in humans [1]. Animal–human transmission occurs primarily through bites or exposure to the saliva of infected animals, with dogs being the main reservoir responsible for most human cases globally [2]. Once transmitted, the virus progresses through the nervous system, leading to fatal neurological symptoms and causing 100% mortality in humans [1, 3]. Rabies may also be transmitted when the saliva of an infected animal comes into contact with human mucous membranes or fresh skin wounds [3].

Rabies affects all mammals and is prevalent worldwide, causing fatality in both humans and animals [1]. WHO estimates that dog-mediated rabies causes 59,000 human deaths annually across 150 countries, with India contributing substantially to this burden [4]. In Viet Nam, rabies incidence has risen sharply in recent years. Animal infections increased from 78 cases in 11 provinces in 2021 to 347 cases across 31 provinces in 2023, while human deaths rose from 69 in 2022 to 82 in 2023 [5]. Alarmingly, during the first quarter of 2024, 27 human deaths and 45 animal cases were reported, indicating a nationwide escalation of the rabies situation [6].

Rabies is preventable through three proven approaches: (1) community awareness, which enables individuals to identify symptoms, seek proper care, and understand post-bite actions; (2) post-exposure prophylaxis using rabies vaccines or rabies immunoglobulin, combined with appropriate wound management, which is nearly 100% effective; and pre-exposure prophylaxis recommended for high-risk groups such as veterinarians; and (3) mass dog vaccination targeting the primary source of human rabies cases. Achieving at least 70% vaccination coverage in dogs can interrupt disease transmission, demonstrating the importance of sustained vaccination efforts [4]. Mass vaccination requires coordination among multiple stakeholders, and pet owners’ participation is critical. Factors such as education level, income, rabies-related knowledge, and access to veterinary services substantially influence whether pets receive active (within the previous 12 months) or inactive (no documented vaccination) rabies immunization [7–10].

In 2015, the WHO set the global goal of “Zero human deaths due to dog-mediated rabies by 2030,” emphasizing the urgency of effective prevention and control strategies. In alignment with this goal, the Prime Minister of Viet Nam issued the national plan for rabies prevention and control (Decision No. 2151-QD-TTg-2021) [11], which aims to achieve 70% vaccination coverage in dogs and cats from 2022 to 2025 and increase the target to 80% from 2026 to 2030.

However, in 2023, national vaccination coverage remained low at 58%, with considerable variation among provinces: only 22 of 63 provinces achieved 70% coverage, 20 reached 50%–69%, and 21 recorded less than 50% [5, 12]. Long An Province, formerly part of Tay Ninh Province until administrative restructuring in 2025, is a large agricultural region in southern Viet Nam with numerous households keeping dogs and cats for companionship or security, many of which are raised in free-range conditions. Between January 2019 and December 2022, the province recorded seven human rabies fatalities and nine confirmed or suspected rabies cases in dogs and cats. Most human deaths were associated with bites from unvaccinated animals, reflecting insufficient awareness and inadequate rabies prevention practices among the population [13].

Despite ongoing national efforts to expand rabies prevention and control, significant gaps persist in high-risk regions such as Duc Hue District in Long An Province. This rural area repeatedly failed to achieve the required 80% annual rabies vaccination coverage before 2021, resulting in persistent human cases and continued transmission risks. Although the long-term campaign “Rabies vaccination for the community” was initiated in 2021 through collaboration among Nong Lam University, Boehringer Ingelheim Viet Nam, and provincial and district veterinary authorities, empirical evidence evaluating its effectiveness remains limited. Existing studies in Viet Nam have largely focused on descriptive vaccination trends or isolated assessments of knowledge, attitude, and practice (KAP), with minimal integration of behavioral analytics, spatial distribution of vaccination coverage, or multivariate determinants influencing proactive vaccination behavior. Critically, no study has provided a data-driven classification of pet owners, particularly owners of Canis lupus familiaris (dogs) and Felis catus (cats), to guide targeted interventions in high-risk communes. After their first appearance, these species are referenced as C. l. familiaris and F. catus, respectively. This lack of integrated evidence limits local authorities’ capacity to strengthen strategies and hampers progress toward the national objective of eliminating dog-mediated human rabies deaths by 2030.

Therefore, this study aims to generate a comprehensive and analytical understanding of rabies control efforts in Duc Hue District during the 2024 campaign. Specifically, the study seeks to (i) summarize the rabies vaccination coverage rate and its demographic and spatial distribution across 11 communes, (ii) identify key factors associated with proactive vaccination behavior among pet owners through multivariate modeling, and (iii) classify owners of C. l. familiaris and F. catus based on their KAP scores using behavioral and statistical segmentation techniques. By addressing these objectives, the study provides essential evidence to support adaptive, community-centered, and data-informed rabies control strategies within the ongoing 2021–2031 vaccination initiative and contributes to Viet Nam’s national goal of achieving “Zero human deaths from dog-mediated rabies by 2030.”

## MATERIALS AND METHODS

### Ethical approval

Ethical clearance was granted by the Faculty of Animal Science and Veterinary Medicine, Nong Lam University, Ho Chi Minh City, Viet Nam, under approval code Agreement_NLU_PCLA_001 (April 2024) and was endorsed by the People’s Committee of Long An Province. The “Confirmation for Data Use and Ethics in Study” was validated by the Sub-Department of Livestock Production, Animal Health, and Aquaculture of Long An (formerly part of the Sub-Department of Agriculture, Tay Ninh Province, as of July 15, 2025), authorizing the use of all collected data for publication.

Before participation, all respondents were informed of the study objectives, the intended data use, and the voluntary nature of their involvement. Oral informed consent was obtained from each participant, which was considered appropriate given the predominantly rural setting and varying literacy levels. The ethics committee reviewed and approved the use of oral consent. All participants were assured anonymity and confidentiality, and no personally identifiable information was collected.

Animal handling during the vaccination campaign followed humane standards recommended by the World Organization for Animal Health and the Department of Animal Health of Viet Nam. No invasive or experimental procedures were conducted beyond routine vaccination. All data were anonymized and securely stored on password-protected institutional servers accessible only to authorized research personnel.

### Study period and location

Long An Province in southern Viet Nam consists of extensive agricultural land and many households that keep dogs and cats for companionship or security, often in free-range environments. Duc Hue District alone has 4,433 households, according to the Sub-Department of Livestock Production, Animal Health, and Aquaculture of Long An in early 2024. The study area encompassed all 11 communes of the district, including five border communes. Field data collection was carried out from April 20 to April 25, 2024.

### Study design

This cross-sectional analytical study was conducted during the 2024 mass rabies vaccination campaign. During vaccine administration, research teams collected general household information, including Global Positioning System (GPS) coordinates, demographic characteristics, and other relevant data. The KAP survey was administered to participating owners following informed consent. Daily data quality control was performed by field coordinators, and the KoboToolbox (https://www.kobotoolbox.org/) form used for data entry was configured with mandatory fields to reduce missing information.

### Conceptual framework

**Figure 1:**
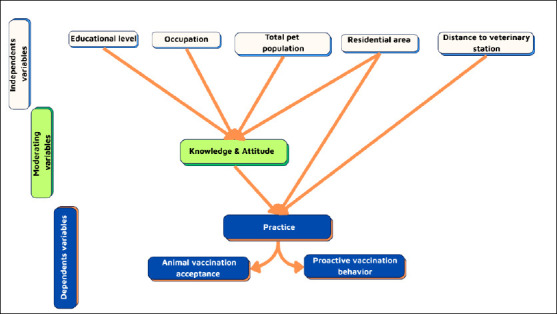
Conceptual framework representing the relationship between socioeconomic variables and practices.

The conceptual framework described the hypothesized relationships between independent variables (educational level, occupation, total pet population, residential area, and distance to the veterinary station), moderating variables, and dependent outcomes (Figure 1). Knowledge and attitude were expected to mediate the relationship between these factors and practice, measured through vaccination acceptance and proactive vaccination behavior. Cluster analysis (CA) was applied to classify household behavioral profiles based on these variables. The CA method appears in this manuscript for the first time, and therefore, the full term is used here; after this point, only “CA” is used.

### Sample size and sampling technique

A general survey was conducted for every vaccinated household, while the KAP survey used a systematic random sampling method with a 1:3 ratio, selecting one in every three vaccinated households. Expected respondent numbers per commune were calculated based on the household distribution and the minimum required sample size. Data on pet-owning households were obtained from the Duc Hue District Veterinary Station using records from the previous year. If a selected household no longer kept dogs, it was replaced by the nearest eligible household. Incomplete KAP data were excluded from the central database.

The minimum sample size of 289 respondents was calculated using Statulator software [14], assuming a 95% confidence interval (CI), an expected 75% satisfactory KAP proportion, and a 5% margin of error. To enhance statistical precision, the final number of collected samples exceeded this minimum, as presented in the Results section.

### Questionnaire development and data collection

The questionnaire consisted of two components:

- General survey: GPS location, demographic characteristics, and household information.
- KAP survey:
- Knowledge: Eight items assessing symptoms, transmission routes, and preventive measures.
- Attitude: Eleven items concerning interactions with unfamiliar dogs.
- Practice: Eight items evaluating rabies prevention behaviors toward humans and animals.

Responses were scored as 1 (correct) or 0 (incorrect or no answer). Maximum possible scores were 8 for knowledge, 11 for attitude, and 8 for practice. The questionnaire was reviewed by two researchers and one veterinary authority, translated into Vietnamese, back-translated into English, and pilot-tested in 3–5 households to ensure clarity and logical flow.

Data collection was conducted concurrently with vaccination activities through direct household interviews. Interviewers received training on KoboToolbox operation and appropriate interview techniques. The application supported offline data collection in remote areas, with data uploaded once internet connectivity was available [15]. Prior to the campaign, households received information on rabies transmission and prevention through leaflets and broadcast announcements. Field coordinators performed daily data quality checks via a hotline support system.

### Data management and statistical analysis

Interview data were exported from KoboToolbox and stored in Microsoft Excel 2021 (Microsoft Corp., Washington, USA). Vaccination coverage was calculated, and all statistical analyses were conducted at a 95% confidence level with significance defined at p < 0.05.

A multivariate logistic regression model assessed associations between demographic variables and proactive vaccination behavior. Independent variables included occupation (five categories), educational level (four categories), total pet population, and distance to the district veterinary station. Preliminary screening at p < 0.2 identified candidate variables for the multivariate model; backward elimination was then applied, retaining variables with p < 0.05. Multicollinearity was evaluated before model finalization.

Internal consistency of KAP scales was assessed using Cronbach’s alpha or Kuder–Richardson 20 for dichotomous items. Due to low internal consistency, a person-centered analytical approach was adopted using principal component analysis (PCA) and CA. The full term PCA appears here for the first time; after this appearance, only “PCA” is used. Responses were used as quantitative variables in PCA and CA to generate behavioral clusters. A hierarchical clustering algorithm initially suggested three clusters (k = 3), which were compared with k = 2 and k = 4. Final cluster selection was based on interpretability, cluster distinctness, and explained variance.

Associations between cluster membership and independent variables (occupation, total pet population, educational level, and distance to veterinary services) were evaluated. Multivariable logistic regression, PCA, and CA were conducted using the lme4 [16] and FactoMineR packages [17] in R Studio version 4.5.1. Spatial mapping was performed using QGIS version 3.36.

## RESULTS

### Distribution of rabies vaccination coverage in Duc Hue District

**Figure 2:**
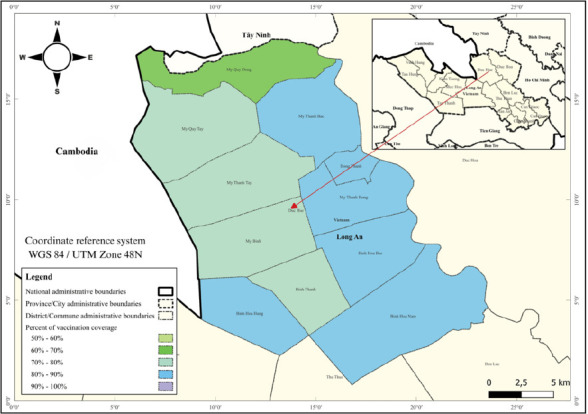
Study area and distribution of rabies vaccination coverage in 2024 across communes of Duc Hue District. Compartments 1–3 represent administrative groupings.

Table 1 and Figure 2 summarize rabies vaccination outcomes across the 11 communes of Duc Hue District. A total of 3,183 pet-owning households were recorded, representing 8,776 pets. During the 2024 campaign, 6,899 pets were vaccinated, resulting in coverage ranging from 60.63% in My Quy Dong (Compartment 2) to 87.78% in Binh Hoa Nam (Compartment 1). Overall, the campaign achieved a vaccination coverage rate of 73.37%, surpassing the national target of 70% established for 2022–2025 [11].

**Table 1:** Summary of vaccination outcomes, calculated based on households and total population, distributed by commune locations in Duc Hue, Long An, 2024.

No.	Communes	Total of pet households	Total of pet population	No. vaccinated pets	No. non-vaccinated pets	Vaccination coverage (%)
1	Binh Hoa Bac^3^	377	850	741	109	87.18
2	Binh Hoa Hung^3^	98	247	212	35	85.83
3	Binh Hoa Nam^1^	214	589	517	72	87.78
4	Binh Thanh^3^	200	538	404	134	75.09
5	Dong Thanh^1^	230	618	499	119	80.74
6	My Binh^3^	145	430	337	93	78.37
7	My Quy Dong^2^	291	983	596	387	60.63
8	My Quy Tay^2^	463	1463	1126	337	76.97
9	My Thanh Bac^2^	231	570	479	91	84.04
10	My Thanh Dong^1^	537	1454	1184	270	81.43
11	My Thanh Tay^2^	397	1034	804	230	77.76
	Total	3183	8776	6899	1877	78.61

1 Commune in compartment 1,

2 Commune in compartment 2,

3 Commune in compartment 3

Figure 2 illustrates the spatial distribution of vaccination coverage across Duc Hue District, Long An Province, Viet Nam. Graduated shading in green, purple, and blue represents different coverage ranges (50% to >100%). Administrative boundaries at national, provincial, district, and commune levels are clearly marked, and each commune is labeled for reference.

### Demographic characteristics of pet owners

Table 2 presents the demographic characteristics of the 1,455 respondents who completed the KAP survey. Most dog and cat owners reported farming as their primary occupation (72.10%), while secondary school was the most common educational level (43.92%). Individuals over 50 years of age represented the largest age group (51.30%), and males comprised 66.32% of respondents. More than half of the surveyed households (54.09%) owned fewer than three pets.

**Table 2:** Social-demographic characteristics of pet owners interviewed for the KAP survey about rabies in 2024 (n = 1455).

Characteristics	Frequency	Percentage
Age <30	48	3.30
30-50	623	42.82
>50	784	53.88
Gender		
Male	965	66.32
Female	490	33.68
Educational levels		
Primary school	435	29.9
Secondary school	639	43.92
High school	304	20.89
Intermediate/College	31	2.13
University	43	2.96
Postgraduate	3	0.21
Occupations		
Authority personnel	65	4.47
Business	143	9.83
Farmer	1049	72.10
Private enterprise officer	23	1.58
Soldier	5	0.34
Others	170	11.68
Number of family members per household		
<3	306	21.03
3-5	982	67.49
>5	167	11.48
Total number of pets per household		
<3	787	54.09
3-5	517	35.53
>5	151	10.38

KAP = Knowledge, attitude, and practice

### Factors influencing proactive vaccination behavior

The logistic regression analysis (Table 3) assessed demographic and logistical variables associated with proactive vaccination behavior among pet owners, using data from 2,807 of the 3,183 surveyed households. Several statistically significant associations were identified.

**Table 3:** Multivariable logistic regression analysis of factors associated with proactive vaccination at veterinary clinics.

Variable	B (Log-odds)	Standard error	OR	95% CI	p-value
Occupation					
Authority person (ref.)					
Farmer	–0.14	0.24	0.87	0.54–1.42	0.58
Solder	0.32	0.88	1.38	0.23–8.38	0.72
Private business	0.19	0.27	1.21	0.79–2.05	0.48
Other	–0.88	0.29	0.41	0.24–0.73	0.002
Education level					
Primary school (ref.)					
Secondary school	0.48	0.11	1.61	1.30–2.01	***
High school	1.03	0.13	2.81	2.19–3.61	***
Postgraduate	1.02	0.25	2.77	1.69–4.49	***
Total pet population	–0.10	0.03	0.91	0.86–0.95	***
Distance (in km)	–0.11	0.01	0.89	0.87–0.91	***
Intercept	−8.33 × 10⁻⁵	0.28	0.99	0.58–1.71	0.99

* p < 0.05, ** p < 0.01,

*** p < 0.001, OR = Odds ratio, CI = Confidence interval

Education level was a strong predictor of proactive vaccination. Compared with owners who completed primary school (reference category), those with secondary school education (odds ratio [OR] = 1.61; 95% CI: 1.30–2.01), high school education (OR = 2.81; 95% CI: 2.19–3.61), or postgraduate education (OR = 2.77; 95% CI: 1.69–4.49) were significantly more likely to exhibit proactive vaccination behavior (p < 0.001).

Occupation also influenced behavior. Individuals categorized under “Other” occupations had significantly lower odds of proactive vaccination (OR = 0.41; 95% CI: 0.24–0.73; p = 0.002) compared with the reference occupation.

Two continuous variables, total pet population per household and distance from the household to the district veterinary station, were significantly negatively associated with proactive vaccination (p < 0.001). Each additional pet was associated with a 9.5% reduction in the odds of proactive vaccination (OR = 0.91; 95% CI: 0.86–0.95). Similarly, each additional kilometer to the district veterinary station was associated with a 10.8% reduction in the odds of proactive vaccination behavior (OR = 0.89; 95% CI: 0.88–0.91).

### Classification of pet owners based on rabies KAP

PCA revealed that the first two principal components accounted for 79.14% of the total variance. CA (Table 4) identified three distinct groups of pet owners based on mean KAP scores.

**Table 4:** Summary of cluster score and classification.

Parameter	Cluster 1	Cluster 2	Cluster 3	The overall mean score in the population
Knowledge	5.72	7.33	7.43	7.03
Attitude	N/A	6.11	9.43	8.72
Practice	4.95	7.26	7.46	6.87
Cluster characteristics	LK^1/^, LP^1/^	HK^1/^, LA^1/^, HP^1/^	HK^1/^, HA^1/^, HP^1/^	

1/ “L” lower than the overall mean score in the population, “H” higher than the overall mean score in the population, “K” knowledge, “A” attitude, “P” practice.

- Cluster 1 comprised individuals with below-average knowledge (5.72) and practice (4.95) scores.
- Cluster 2 included participants with above-average knowledge (7.33) and practice (7.26) scores but a below-average attitude score (6.11).
- Cluster 3 demonstrated the strongest overall performance, with above-average knowledge (7.43), attitude (9.43), and practice (7.46) scores.

Cluster composition further highlighted several demographic patterns (Table 5).

- Cluster 1 primarily consisted of individuals with primary school education and residents of Compartments 1 and 2 (324 participants; 22.27%).
- Cluster 2 (239 participants; 16.43%) was mostly composed of residents of Compartment 2, individuals with middle school education, and households owning fewer than three pets.
- Cluster 3 was the largest cluster (892 participants; 61.31%), mainly including residents from Compartment 1, individuals with high school or intermediate/college education, and those engaged in farming.

**Table 5:** Significant variables and the number of participants contributing to each cluster.

Cluster	Significant categorical variables	V-test	Number of participants in each cluster (n = 1455)
Cluster 1	Primary school level	6.32	324 (22.27%)
	Communes in compartment 1	3.10	
	Communes in compartment 2	2.04	
Cluster 2	Middle school level	2.55	239 (16.43%)
	Communes in compartment 2	13.2	
	Number of owned pets <3	2.37	
Cluster 3	Farmer	2.98	892 (61.31%)
	Communes in compartment 1	5.17	
	High school level	2.74	
	Intermediate/college level	2.13	

## DISCUSSION

### Evaluation of rabies vaccination coverage rate

The 2024 vaccination campaign achieved an overall coverage rate of 78.61%, representing a slight decline from the 81% coverage recorded in 2023 [18]. Despite this reduction, the campaign successfully exceeded the 70% coverage level recommended by WHO [4]. My Quy Dong Commune reported the lowest coverage (56.03%), a level not observed in the preceding four years [18]. This unusually low coverage may be attributed to inadequate allocation of human resources, including vaccination volunteers and local guides, particularly in this commune, which had the largest pet population. The observed year-to-year variability underscores the continued need to strengthen logistical and operational capacity, a critical determinant of the effective implementation of mass vaccination programs, especially in areas with large numbers of owned pets [19].

### Factors influencing proactive vaccination at veterinary clinics

This study identified several demographic and contextual variables influencing proactive vaccination behavior among pet owners. Education level was a particularly strong predictor, with individuals who attained at least secondary education demonstrating a substantially higher likelihood of seeking vaccination services for their animals. This finding aligns with previous studies showing that higher educational attainment is associated with greater knowledge of rabies and preventive practices [20, 21].

Employment status also influenced vaccination behavior. Specifically, individuals classified under “Other” occupations exhibited significantly lower proactive vaccination tendencies. These results underscore the importance of tailoring rabies communication and outreach strategies to different social and occupational subgroups to improve vaccination uptake.

Distance to veterinary services and total household pet population were also significant determinants of vaccination behavior. Limited access to veterinary establishments has been previously recognized as a barrier to achieving high vaccination coverage [20]. Households with more pets may face greater financial, logistical, or time constraints, reducing the likelihood of presenting animals for vaccination. These findings highlight the need to improve service accessibility, such as through mobile vaccination units and door-to-door campaigns, to sustainably increase vaccination coverage in rural and underserved communities.

### Cluster formation and associated behavioral characteristics

CA identified three distinct household groups based on KAP performance. Cluster 1 represented individuals with low Knowledge and Practice scores and consisted mainly of residents in Compartments 1 and 2 with primary school education. This group would benefit from targeted educational interventions at the elementary level to improve rabies awareness and preventive behaviors.

Cluster 2 exhibited relatively strong Knowledge and Practice scores but weaker attitude scores, suggesting gaps in confidence, risk perception, or behavioral engagement despite an understanding of rabies prevention. This group may require targeted behavioral messaging or community-based reinforcement strategies.

Cluster 3, the largest group, demonstrated strong performance across all KAP domains and consisted predominantly of individuals residing in Compartment 1 with high school or intermediate/college education and engaged in farming. This group showed favorable awareness and behaviors toward rabies vaccination.

Comparison with findings from the previous three years indicates notable improvement across some formerly low-performing groups. For example, individuals residing in border communes (Compartment 2) and farmers, groups previously associated with weak KAP outcomes [18], did not emerge as distinct low-performing clusters in 2024. This shift suggests that sustained educational outreach and community engagement have yielded positive behavioral changes.

However, the primary determinants of proactive behavior in 2024 were more strongly associated with distance to veterinary services and total household pet population, indicating that logistical and structural barriers remain substantial challenges. Without continued support, such as annual vaccination campaigns or improved veterinary infrastructure, these barriers may reduce coverage consistency over time.

### Study limitations

Several limitations should be considered when interpreting the findings of this study. First, the cross-sectional design limits causal inference about the association between factors and vaccination outcomes. Second, KAP responses were self-reported, which may introduce recall bias or social desirability bias. Third, the sample included only households participating in the vaccination campaign, potentially underrepresenting unvaccinated households and associated risk factors. Finally, the study did not employ a validated KAP measurement scale tailored to rural Vietnamese populations. Due to these limitations, KAP outcomes were interpreted cautiously, with emphasis placed on population-level behavioral clustering rather than linear score constructs.

### Implications and One Health relevance

This study, to the best of our knowledge, is the first to apply PCA–based household segmentation to categorize pet owners’ KAP profiles in the context of rabies control in southern Vietnam. By integrating demographic factors with behavioral characteristics, the study provides a more nuanced understanding of heterogeneity among pet owners and identifies groups most in need of targeted interventions.

The integrated approach, combining spatial mapping, multivariate modeling, and behavioral segmentation, aligns with the One Health framework of the ASEAN Rabies Elimination Strategy [22]. These findings can support provincial and national rabies control programs by identifying high-risk behavioral clusters before campaign implementation and allocating resources more efficiently. Strengthening educational programs for primary school–level audiences, enhancing veterinary service accessibility, and leveraging community leaders and peer networks may further improve rabies vaccination uptake and support sustainable rabies elimination efforts [7, 23].

## CONCLUSION

The 2024 mass rabies vaccination campaign in Duc Hue District achieved a coverage rate of 78.61%, surpassing the national target of 70% and demonstrating substantial progress in controlling rabies transmission among C. l. familiaris and F. catus. However, coverage varied notably across communes, with My Quy Dong reporting a markedly low level of 56.03%. Multivariate analysis identified education level, occupation, total household pet population, and distance to veterinary services as significant predictors of proactive vaccination behavior. Furthermore, PCA–based cluster classification revealed three distinct behavioral groups with varying strengths in KAP, emphasizing the need for tailored communication and intervention strategies.

A major strength of this study is its integrated analytical approach, which combined spatial mapping, logistic regression modeling, and CA to provide a comprehensive understanding of community-level vaccination behaviors. By producing the first empirical classification of pet owners’ KAP profiles in southern Viet Nam, this study offers a data-driven foundation for more precise targeting of resources, awareness programs, and vaccination logistics. The use of digital tools for real-time data collection and standardized field monitoring further enhances the operational relevance and reliability of the findings.

Looking ahead, future research should include unvaccinated households to identify additional behavioral and structural determinants influencing vaccine uptake. Longitudinal monitoring is needed to evaluate how KAP profiles evolve over time, particularly after the withdrawal of external support from annual vaccination campaigns. Developing and validating a culturally adapted KAP assessment tool for rural Vietnamese contexts could improve measurement accuracy and enhance comparative evaluations across regions. Additionally, integrating real-time geographic information systems and deploying mobile vaccination units may increase efficiency in remote or high-risk communes.

In conclusion, this study underscores the importance of sustained community engagement, expanded access to veterinary services, and data-informed planning to achieve long-term rabies control. By aligning with the One Health framework and Viet Nam’s national strategy for “Zero human deaths due to dog-mediated rabies by 2030,” these findings provide actionable insights for refining vaccination strategies and strengthening rabies elimination efforts in Duc Hue District and other high-risk regions.

## DATA AVAILABILITY

The supplementary data can be made available from the corresponding author upon request.

## AUTHORS’ CONTRIBUTIONS

LPBT, BDT, and TQL: Conceived and designed the study. DTKN, NYLN, TNN, TAL, TPP, MDV, KTVD, OTKV, KTMN, KMTB, and TNV: Conducted field surveys and designed and aided data collection. DTKN, NYLN, TNN, TAL, and TPP: Data analysis and drafted the manuscript. OTKV, KTMN, KMTB, and TNV: Interpreted the results. LPBT, BDT, TQL, MDV, and KTVD: Edited the manuscript. All authors have read and approved the final version of the manuscript.
